# Complex Emotion Recognition via Facial Expressions with Label Noises Self-Cure Relation Networks

**DOI:** 10.1155/2023/7850140

**Published:** 2023-01-17

**Authors:** Xiaoqing Wang, Yaocheng Wang, Deyu Zhang

**Affiliations:** ^1^Shenyang Ligong University, Shenyang 110168, China; ^2^Key Laboratory of Information Network and Information Countermeasure Technology of Liaoning Province, Shenyang Ligong University, Shenyang 110168, China

## Abstract

Current deep learning-based facial expression recognition mainly focused on the six basic human emotions and relied on large-scale and well-annotated data. For complex emotion recognition, such a large amount of data are not easy to obtain, and a high-quality annotation is even more difficult. Therefore, in this paper, we regard complex emotion recognition via facial expressions as a few-shot learning problem and introduce a metric-based few-shot model named self-cure relation networks (SCRNet), which is robust to label noises and is able to classify facial images of new classes of emotions by only few examples from each. Specifically, SCRNet learns a distance metric based on deep features abstracted by convolutional neural networks and predicts a query image's emotion category by computing relation scores between the query image and the few examples of each new class. To tackle the label noise problem, SCRNet gives corrected labels to noise data via class prototype stored in external memory during the meta-training phase. Experimenting on public datasets as well as on synthetic noise datasets demonstrates the effectiveness of our method.

## 1. Introduction

Emotion recognition via facial expressions plays a very important role in human-computer interaction and intelligent medical treatment. The major task of facial expression recognition (FER) is to classify facial images into certain predefined emotion categories. Most of the facial expression datasets, such as CK+ [1], MMI [2], SFEW [3], and FER-2013 [4], provide facial image annotations according to the six basic human emotions introduced by Ekman et al., including happiness, surprise, sadness, fear, disgust, and anger [5]. However, human emotions are complicated, and subsequent research has shown that a larger number of emotion categories exist in human facial expressions [6]. Therefore, a simple classification model with basic emotion categories cannot well reflect people's feelings in real life. However, the annotation of complex human emotions, which is essential for supervised machine learning, is a highly psychological professional work. Therefore, compared with the six basic emotions, datasets that provide more complex emotion labels are rare. RAF-DB [7], for example, contains about thirty thousand facial expression samples with six-class basic emotions, whereas it only provides about four thousand samples with 11-class complex emotions. Such a small number of samples in these complex emotion categories cannot meet the needs of training a convolutional neural network (CNN) [8], which is a common algorithm for facial expression recognition tasks these years. Focusing on this problem, we propose to treat complex facial expression recognition as a meta-learning task. During the meta-training process, we utilize the basic expressions to simulate the situation of few-shot classification and train the model to obtain the ability to distinguish emotions given only a small amount of data, so as to achieve the purpose of complex emotion classification in the test phase using only a small amount of data.

Another problem with facial expression recognition is the training data uncertainties. There are two ways of datasets collection for FER, that is, “lab-controlled” and “in the wild.” Widely used FER datasets such as JAFFE, CK+, and MMI [1, 9–11] are “lab-controlled” datasets, the data of which are collected by inducing volunteers to make corresponding expressions according to the emotion prompts. The annotations of these datasets are trustworthy but the facial expressions are less natural. FER datasets “in the wild,” such as FER-2013, RAF-DB, and SFEW [4, 12], collect natural human facial images from the internet or films in the first place and then ask experts or volunteers to give these samples emotion labels. Compared with the “lab-controlled” scenario, FER in the wild is more practical but also more challenging, because samples collected in the wild differ from illumination, resolution, and background, and may suffer from subjective annotations. Training with these data uncertainties, especially the label noises, will do harm to the meta-learning process. To address this issue, we propose a self-cure relation net (SCRNet), which can suppress the label uncertainties in the meta-learning process.

Our main contributions to this study are as follows:

We expand the facial expression recognition from the six basic emotion categories to more complicated and compound emotions. Considering the fact that annotation for complex facial expressions is a highly professional work and large datasets are hard to get, we propose to formalize this task as a few-shot meta-learning problem.

Data uncertainties such as label noises will do harm to the meta-training process; therefore, we propose a self-cure relation net (SCRNet). This few-shot learning model will detect label noises during the training process and give these images corrected labels based on class prototypes.

## 2. Related Work

### 2.1. Facial Expression Recognition

In the broad scene, FER includes the following technical steps: facial image preprocessing (e.g., face detection and face alignment), feature extraction, and feature classification, among which the feature extraction is the most vital part. According to the feature extraction strategies, current FER methods can be grouped into two categories: predesigned feature-based methods and learned feature-based methods. Predesigned feature-based methods mainly focus on low-level features such as textures and edge distributions. The most commonly used predesigned features are local binary patterns (LBP) [13], histogram of oriented gradients (HoG) [14], scale-invariant feature transform (SIFT) [15, 16], and so on. As for the learned features, methods based on deep supervised learning are the most effective at present. Such algorithms usually use a CNN as the feature extractor, such as VGG [17], ResNet [18], and Mobilenet [19–21]. Ideally, with sufficient high-quality labeled samples and many iterations of training, the CNNs can learn to extract high-level representations that build from low-level features. Many studies have shown that, for the basic emotion recognition, the facial expression features learned by CNNs outperform predesigned features and have achieved state-of-the-art performances [22, 23].

Compared with basic emotion recognitions, the studies with complex facial expression recognitions are less concerned. Li et al. [7] constructed a database RAF-DB that contains compound expressions in the wild and proposed DLP-CNN. To address the ambiguity and multimodality of facial expressions in the wild, they introduce a locality-preserving loss. Liang et al. [24, 25] proposed different strategies to divide facial expressions with fine-grained emotions and proposed classification algorithms accordingly. Current supervised deep-learning FER methods need large-scaled and well-labeled training datasets which can be hard to get for complex facial expression. The lack of samples will hinder the deep models to generalize, resulting in overfitting.

### 2.2. Few-Shot Learning

Few-shot learning methods are designed to learn how to recognize a new class of samples with limited data. Vinyals [26] introduced a matching network that illuminated the concept of an episode-based strategy means detailing the task from mini-batch to mini-batch. The prototypical network was introduced by Snell et al. [27] to compute the distance between a single prototype representation and each class. In the embedding space, the prototype of one class presents the mean of its support set. Finding the nearest class prototype is the most necessary condition to classify the embedded query. Sung [28] proposed a relation network that learned a deep distance metric during meta-learning and then classified the new classes with few samples by computing relation scores between it and query images. Simon [29] introduced dynamic classifiers with a subspace method into few-shot learning. They calculate a subspace of feature space for each category, then project the feature vector of the query sample into the subspace, and predict its category by measuring the distance in the subspace. Zhu et al. [30] utilize a relation network to recognize the facial expressions with insufficient samples.

### 2.3. Learning with Uncertainties

FER in the wild constantly faces the problem of data uncertainties, including low-quality or occluded images, ambiguous facial expressions, and noisy labels. Zeng et al. [31] propose a framework to leverage the annotation errors and bias between different FER datasets. Wang et al. [32] introduce a region attention network (RAN) that is occlusion robust for FER, and Wang [33] designs a self-cure network (SCN) to suppress the label uncertainties in FER datasets.

## 3. Methodology

### 3.1. Problem Definition

**Figure 1:**
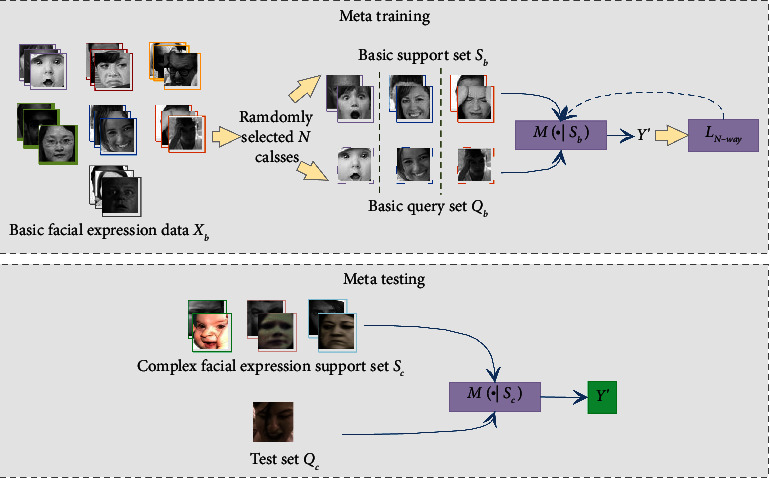
Meta-learning process for complex facial expression recognition.

Basic facial expression recognition task is often considered as a supervised image classification task. In such image classification tasks, training and testing samples are all from the same group of categories, and a large amount of well-annotated data are essential. As we can see from Table 1, the number of samples of the six basic emotions is relatively large. However, as shown in Table 2, the number of complex expression samples is small. Such a small number of samples are insufficient to train a deep classification model from scratch, and simple transfer learning will result in overfitting. The expression categories of basic expression and complex expression are different, but they have certain similarities in feature characteristics. Moreover, the basic expression dataset is relatively large, which is suitable for the training process of meta-learning to make the model acquire the ability to learn. Therefore, instead of using complex expression data to train an emotion classification model directly, we summarize the recognition of complex expressions as a few-shot meta-learning problem.  Figure 1 illustrates the meta-learning process of complex facial expression recognition. The meta-learning process has two parts: the meta-training and the meta-testing. During the training phase, only basic expression data is used, and the small amount of complex expression data only participated in the testing phase.

In the meta-training stage, we divide the training phase into multiple episodes in order to imitate the situation of a small number of labeled samples. Each episode is an *N*-way *K*-shot experiment setting. We select *N* classes from the 7 classes of basic emotions, and for each class, *K*-labeled samples are selected as a priori knowledge, which is called the basic support set, denoted as *S*_*b*_={(*x*_*i*_, *y*_*i*_)}_*i*=1_^*m*=*K*×*N*^, where *x*_*i*_ denotes the image, and *y*_*i*_ is the corresponding label. We also selected *v* samples from these same *N* classes, called the basic query set, *Q*_*b*_={(*x*_*i*_, *y*_*j*_)}_*j*=1_^*v*^, where *x*_*j*_ denotes the image, and *y*_*j*_ is the corresponding label. The model *M* abstract image features and classifies the query set samples into one of the *N* classes according to feature similarities between the query and support set. The optimization goal of the model *M* is to minimize the classification accuracy of the query set conditioned on the support set. In this way, through enough episodes of training, the model *M* can master the ability to classify human expressions given only a limited annotated support samples.

In the meta-testing stage, we give model *M* a small support set *S*_*c*_, containing *N* classes of complex expressions that have never participated in the training stage. The model *M* trained by meta-learning will classify the test samples *Q*_*c*_ into one of the *N* classes of complex emotions.

### 3.2. Proposed Method

#### 3.2.1. Meta-Learning Model Overview

**Figure 2:**
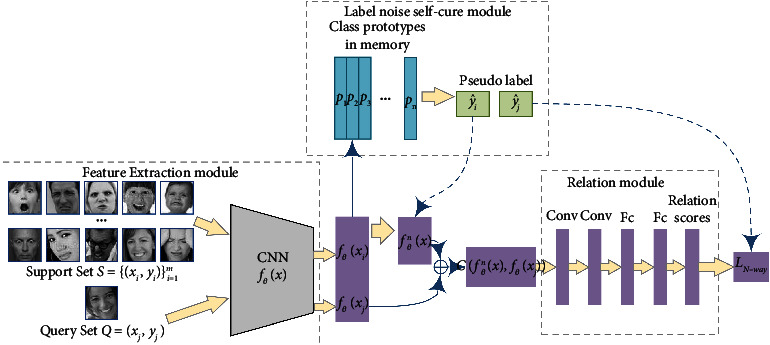
Architecture of meta-learning model SCRNet.

The architecture of our meta-learning model for complex FER is a metric-based few-shot learning network called label noise self-cure relation net (SCRNet). As is shown in Figure 2, it has three major parts: (1) feature extraction module, (2) relation module, and (3) label noise self-cure module.

For the feature extraction module, we use CNN as the feature extractor *f*_*θ*_, mapping input image *x* into a feature space, generation of feature maps *f*_*θ*_(*x*). As for the relation module, we use a relation model *g*_*φ*_ inspired by [28] to measure the distance between query sample features and support sample features, and the category of a query sample is predicted based on the relation score. The self-cure module is designed to reduce the impact of label noise during the meta-training process. We calculate the averaged feature maps of each class as the class prototype, and by comparing the similarity between the feature map of an input image *x* with class prototypes *P*, the self-cure module will generate a corrected label $\hat{y}$ for the input image *x*.

The training of the proposed framework contains two phases: the first phase is to warm up and initialize the feature extraction network with the original noisy label and the second phase is to train the network with the self-cure module. During the second phase, we calculate and update class prototypes based on the network trained in the first stage. These prototypes are used to generate the corrected label.

#### 3.2.2. Model Initialization

We first train the feature extractor and relation module with no label correction for some episodes to warm up the framework and initialize the training class prototypes. In this phase, for each class *nϵN* in the support set, we first calculate the averaged feature map:

(1) $$\begin{array}{c} f_{\theta }^{n}\left(x_{i}\right)=\underset{y_{i}=n}{avg}\left\{f_{\theta }\left(x_{i}\right)\right\}\text{.} \end{array}$$

Then, we combined *f*_*θ*_^*n*^(*x*_*i*_) with the query sample feature map *f*_*θ*_(*x*_*j*_) in-depth into a concatenated feature *C*(*f*_*θ*_^*n*^(*x*_*i*_), *f*_*θ*_(*x*_*j*_)). We fed *C*(*f*_*θ*_^*n*^(*x*_*i*_), *f*_*θ*_(*x*_*j*_)) into the relation module *g*_*φ*_ to generate relation scores *r*_*n*,*j*_, which indicate the similarities between the query image and class *n*.

The training loss of the warm up phase is calculated and optimized as follows:

(2) $$\begin{array}{c} L_{N-way}\leftarrow \text{argmin}{\overset{N}{\underset{n=1}{\sum }}\overset{v}{\underset{j=1}{\sum }}\left(r_{n,j}-\left(y_{j}==n\right)\right)}^{2}\text{.} \end{array}$$

#### 3.2.3. Meta-Training with Label Noise Self-Cure Module

Ideally, through enough episodes of meta-training, the model should be able to obtain the ability to extract the emotional features which have high interclass variations. However, because the training process of our few-shot learning algorithms only uses a small number of samples at a time, the existence of label noises will make it difficult for the model to find appropriate high-level features for facial expressions and turn to find some accidental differentiation or low-level features instead. In other words, the existence of training label noises may affect the feature learning direction of the model and then affect the convergence speed and classification accuracy. Therefore, in the meta-training stage, it is necessary to take into consideration of the label noise. After the warm up phase, training episodes with label correction are conducted to further optimize the model parameters. The process of a training episode with a self-cure module is illustrated in Algorithm 1.

Given an image *x*, we calculate the cosine similarity between extracted features and prototypes and obtain the corrected label $\hat{y}$ for each sample in *S*_*b*_ and *Q*_*b*_ by

(3) $$\begin{array}{c} \hat{y}={argmax}_{n}\cos\;\left(f_{\theta }\left(x\right),p_{n}\right),n=1,2,\ldots ,N\text{.} \end{array}$$

Taking the corrected label into consideration, we calculate the averaged feature map of the support set using

(4) $$\begin{array}{c} f_{\theta }^{n}\left(x\right)=\left(1-\alpha \right)\times \underset{y_{i}=n}{avg}\left\{f_{\theta }\left(x_{i}\right)\right\}+\alpha \times \underset{{\hat{y}}_{i}=n}{avg}\left\{f_{\theta }\left(x_{i}\right)\right\}\text{.} \end{array}$$

We update and store the prototypes of each class in memory. The prototype of the class *nϵN* is calculated and updated as follows:

(5) $$\begin{array}{c} p_{n}⟵\frac{1}{K+1}\left(p_{n}+\underset{\left(x_{j},y_{j}\right)\in S_{n}}{\sum }f_{\theta }\left(x_{i}\right)\right)\text{,} \end{array}$$

where *S*_*n*_ denotes a subset of the support set *S*_*b*_ containing samples (*x*_*i*_, *y*_*i*_) that *y*_*i*_=*n* *nϵN*.

And the train loss in this phase is calculated and optimized as follows:

(6) $$\begin{array}{c} L_{N-way}⟵\text{argmin}{\overset{N}{\underset{n=1}{\sum }}\overset{v}{\underset{j=1}{\sum }}\left(r_{n,j}-\left(1-\alpha \right)\left(y_{j}==n\right)-\alpha \left({\hat{y}}_{j}==n\right)\right)}^{2}\text{,} \end{array}$$

where *α* is the balance ratio of the original label and the corrected label.

## 4. Experiments

### 4.1. Datasets

**Figure 3:**
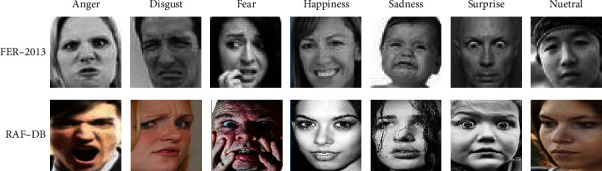
Examples of facial expression images from the six basic emotions.

**Figure 4:**
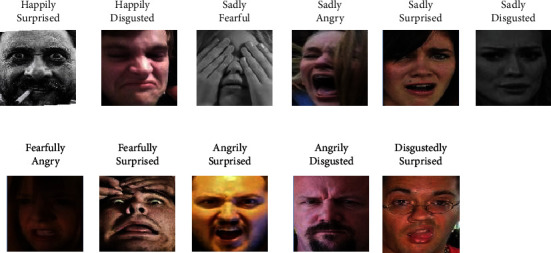
Examples of facial expression images from RAF-comp.

We evaluate our method proposed in this paper using two well-known “in the wild” FER datasets: FER-2013 and RAF-DB. Tables 1 and 2 give the number of images per class used in our experiments. Figures 3 and 4 show some of the example images in these datasets.

#### 4.1.1. FER-2013

The FER-2013 was originally collected from the Internet and brought out as a benchmark in the ICML 2013. The training set of this dataset contains 28,709 images, which are labeled with seven emotion categories (the six basic emotions and neutral).

#### 4.1.2. RAF-DB

The real-world affective face database (RAF-DB) consists of two parts. The first part contains 15339 images with the same seven labels as FER-2013, which was referred as RAF-basic in the follow-up experiments. The second part of this dataset contains 3954 images annotated with eleven compound emotion labels, which is referred as RAF-comp.

### 4.2. Implementation Details

For feature extraction, we choose ResNet18 as the backbone. As for the relation module, we use a structure that has two convolution blocks followed by two full connection layers. The relation scores' output by the relation module indicates the similarities between the query image and all the categories of support samples.

In the meta-training process, all the facial images are resized to 224 × 224 pixels, and data augmentations including flipping and color jitter are applied. The training episodes are set as 30000 (3000 episodes for warm up phase, and 27000 episodes for the self-cure phase), and each episode represents a 5-way 5-shot classification task. The balance ratio *α* is set as 0.4. Adam optimizer with an initial learning rate of 10^−3^ is used. All experiments are conducted under the environment of Ubuntu 18.04, PyTorch 1.7.1 on NVIDIA 3080 GPU.

### 4.3. Results and Discussion

#### 4.3.1. Performance Evaluation

In our experiments, we want to evaluate that with only few samples, how well can our model recognize complex emotions that are never seen in the training phase. Therefore, we use RAF-basic or FER-2013 as the base classes for training and RAF-comp as the new classes for testing and refer to them as RAF-basic ⟶ RAF-comp and FER-2013 ⟶ RAF-comp, respectively.

**Figure 5:**
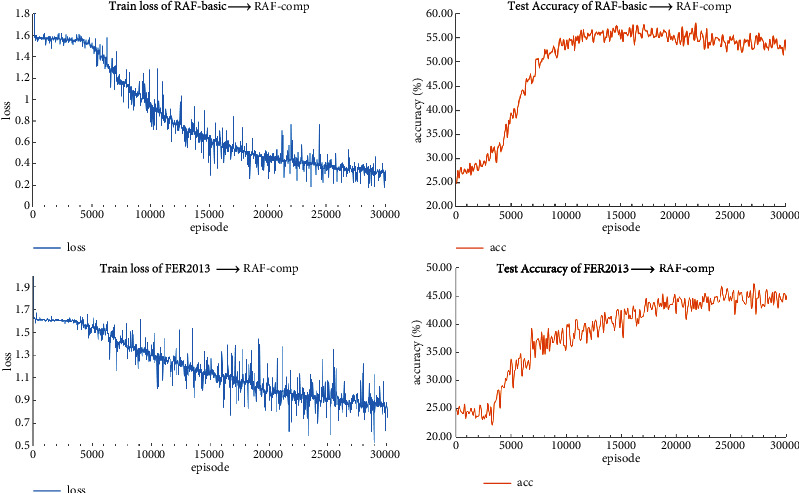
Train loss and test accuracy.

In this experiment, we record the train loss every 10 episodes and test the recognition accuracy with 100 testing episodes after every 100 training episodes and record the averaged test results. From Figure 5, we can see that, in both experiment settings, the train loss decreases faster with obvious vibration after 3000 episodes when the proposed self-cure module is introduced in the training phase. The rather obvious loss vibration is because we constantly update class prototypes during the training process, resulting in the changes of the corrected labels. We can also see that the recognition accuracy of the model trained on RAF-basic is higher than that trained on FER-2013. This is because RAF-basic and the test dataset RAF-comp are collected and annotated by the same research group, whereas FER-2013 and RAF-comp have a larger domain shift.

#### 4.3.2. Robustness on Label Noises

In this part of the experiment, we randomly choose 2%, 5%, 10%, 20%, and 30% of the samples in the training set and change their labels randomly. Then, we train the model with and without the proposed self-cure module to test the robustness of our method with training uncertainties.

**Figure 6:**
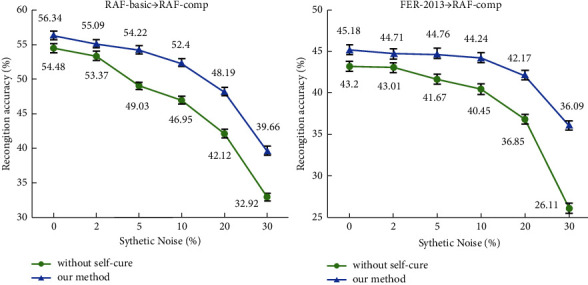
Recognition accuracy with synthetic noise.

As shown in Figure 6, under both RAF-basic ⟶ RAF-comp and FER-2013 ⟶ RAF-comp experiments settings, when there is no synthetic label noise, the test results with the self-cure module are slightly higher than those that without the self-cure module. This is because the original facial expression training data cannot be ideal and inevitably contains some label noises. When the percentage of synthetic noise gets higher, the effectiveness of the proposed self-cure module becomes more evident. Although suffering from the synthetic noise, the recognition accuracy of our method drops slower with the self-cure module applied.

**Figure 7:**
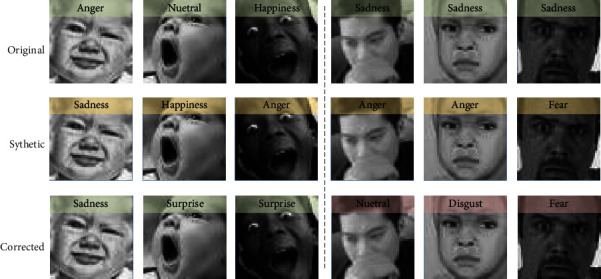
Label comparison of the original labels, synthetic labels, and correction failures.

As shown in Figure 7, the images on the first row are with the original labels. And the images on the second row are with the synthetic labels we changed randomly. These synthetic labels take part in the training in the warm up phase and are corrected during the training with the label self-cure model. The third row shows the corrected labels after 20000 episodes. Some of the corrected labels are not the same as the original labels but are more reasonable, such as the left three columns in Figure 7. On the other hand, for hard examples with occlusions or difficult to distinguish even for human (see the right three columns in Figure 7), the self-cure module fails to give suitable corrections. The existence of these corrected labels that are inconsistent with the original labels may affect the convergence speed and accuracy of the model.

#### 4.3.3. Studies with the Hyper-Parameter *α*

**Figure 8:**
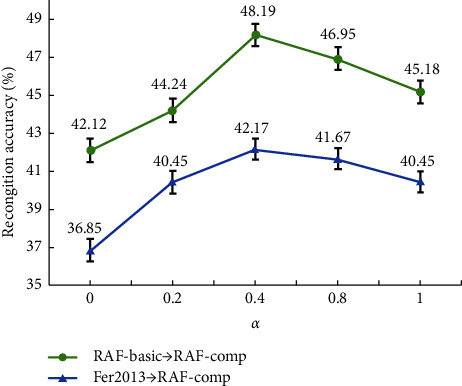
Effects of balance ratio *α*.

The balance ratio *α* decides how much will the network rely on the corrected labels. If *α*=0, the model will take no consideration of the corrected label during the training phases whereas when *α*=0, the training will fully depend on the corrected labels. We test the recognition accuracy under 20% synthetic noise with different *α* to discover its influence. As shown in Figure 8, we can see that although we assume that the corrected labels are beneficial for the emotion recognition training, the model trained solely on the corrected label does not perform the best. The model achieves the best performance when *α*=0.4, trained by the original and the corrected label jointly. The result proves our previous discovery that the self-cure module has difficulties with hard example corrections and may misidentify some samples as noise. Therefore, directly replacing all labels with corrected labels will reduce the generalization ability of the model.

#### 4.3.4. Comparison with Other Methods

To further evaluate our method, we compare it with both predesigned feature-based methods and few-shot meta-learning methods. For a fair comparison, the other meta-learning methods also use ResNet18 for feature embedding, and the architecture of the relation model for relation net and our method are the same. The comparison experiments are also conducted under the two scenarios discussed previously, and for all the few-shot learning methods, we calculate the average accuracy of 600 testing episodes. The results in Table 3 show that, without the proposed self-cure module, our method can achieve similar accuracy compared with the state-of-the-art meta-learning method and outperform other handmade feature methods. When trained with the self-cure module, our method outperforms all the other methods.

## 5. Conclusions

In this paper, we focused on the task of complex emotion recognition via facial expressions. We propose to use a few-shot meta-learning framework to treat complex facial emotion recognition, which can solve the problem of lack of annotations. We introduce a metric-based few-shot network called self-cure relation net (SCRNet), which can suppress the label uncertainties in the meta-learning process. Experiments on public datasets show that our SCRNet is robust to label noise and obtain state-of-the-art performance compared with other few-shot learning methods.

## Figures and Tables

**Algorithm 1:**
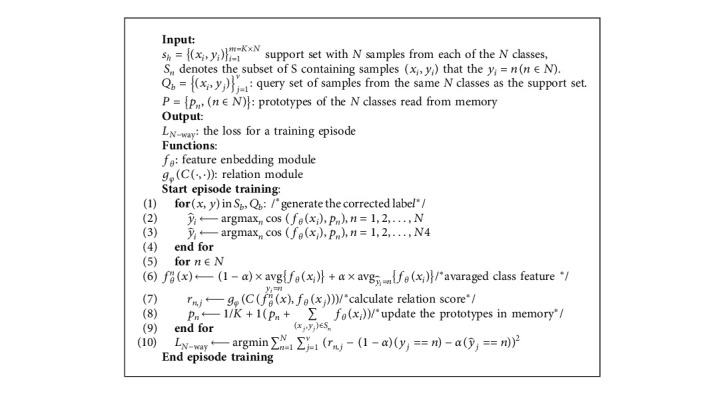
Loss computation for a training episode.

**Table 1:** The number of samples per class of the basic emotion categories in FER-2013 and RAF-DB.

Datasets	Happiness	Surprise	Sadness	Fear	Disgust	Anger	Neutral
FER-2013	4406	1962	2978	2510	269	2428	3038
RAF-DB	5957	1619	2460	355	877	867	3204

**Table 2:** The number of samples per class of compound emotion categories in RAF-DB.

Happily surprised	Happily disgusted	Sadly fearful	Sadly angry	Sadly surprised	Sadly disgusted	Fearfully angry	Fearfully surprised	Angrily surprised	Angrily disgusted	Disgustedly surprised
697	266	129	163	86	738	150	560	176	841	148

**Table 3:** Recognition accuracy compared with other methods.

Method	Self-cure module	RAF-basic ⟶ RAF-comp (%)	FER-2013 ⟶ RAF-comp (%)
LBP^*∗*^		45.51	—
HOG^*∗*^		51.89	—
Gabor^*∗*^		53.54	—
Relation net		54.48	43.10
Prototypical net		55.08	43.22
SCRNet	×	55.09	43.10
SCRNet	✓	56.34	45.18

^*∗*^Experiment results produced by [7].

## Data Availability

The datasets used during the current study are available in the following repository: https://www.kaggle.com/c/challenges-in-representation-learning-facial-expression-recognition-challenge. http://www.whdeng.cn/raf/model1.html.
